# Online Activity and Participation in Treatment Affects the Perceived Efficacy of Social Health Networks Among Patients With Chronic Illness

**DOI:** 10.2196/jmir.2630

**Published:** 2014-01-10

**Authors:** Racheli Magnezi, Yoav S Bergman, Dafna Grosberg

**Affiliations:** ^1^Department of Public Health and Health Systems Management ProgramBar Ilan UniversityRamat GanIsrael; ^2^Gertner Institute for Epidemiology and Health Policy ResearchSheba Medical Center, Tel HashomerTel AvivIsrael; ^3^Interdisciplinary Department for Social SciencesBar Ilan UniversityRamat GanIsrael

**Keywords:** Internet, social health network, Patient Activation Measure (PAM), online health network

## Abstract

**Background:**

The use of online health-related social networks for support, peer-to-peer connections, and obtaining health information has increased dramatically. Participation in an online health-related social network can enhance patients’ self-efficacy and empowerment, as they are given knowledge and tools to manage their chronic health condition more effectively. Thus, we can deduce that patient activation, the extent to which individuals are able to manage their own health care, also increases. However, little is known about the effects of participation in online health-related social networks and patient activation on the perceived usefulness of a website across disease groups.

**Objective:**

The intent of the study was to evaluate the effects and benefits of participation in an online health-related social network and to determine which variables predict perceived site usefulness, while examining patient activation.

**Methods:**

Data were collected from “Camoni”, the first health-related social network in the Hebrew language. It offers medical advice, including blogs, forums, support groups, internal mail, chats, and an opportunity to consult with experts. This study focused on the site’s five largest and most active communities: diabetes, heart disease, kidney disease, spinal injury, and depression/anxiety. Recruitment was conducted during a three-month period in which a link to the study questionnaire was displayed on the Camoni home page. Three questionnaires were used: a 13-item measure of perceived usefulness (Cronbach alpha=.93) to estimate the extent to which an individual found the website helpful and informative, a 9-item measure of active involvement in the website (Cronbach alpha=.84), and The Patient Activation Measure (PAM-13, Cronbach alpha=.86), which assesses a patient’s level of active participation in his or her health care.

**Results:**

There were 296 participants. Men 30-39 years of age scored higher in active involvement than those 40-49 years (*P*=.03), 50-64 years (*P*=.004), or 65+ years (*P*=.01). Respondents 20-29 years of age scored higher in perceived usefulness than those 50-64 years (*P*=.04) and those 65+ years (*P*=.049). Those aged 20-29 years scored significantly lower on the PAM-13 scale than those aged 30-39 years (*P*=.01) and 50-64 years (*P*=.049). Men and women had similar PAM-13 scores (*F*_9,283_=0.17, *P*=.76). Several variables were significant predictors of perceived usefulness. Age was a negative predictor; younger age was indicative of higher perceived usefulness. Active involvement was a positive predictor. There was a negative relationship found between PAM-13 scores and perceived usefulness, as taking a less active role in one’s own medical care predicted higher perceived website usefulness. A trend toward higher frequency of website activity was associated with increased perception of usefulness.

**Conclusions:**

Online health-related social networks can be particularly helpful to individuals with lower patient activation. Our findings add information regarding the social and medical importance of such websites, which are gradually becoming an inseparable part of day-to-day chronic disease management in the community.

## Introduction

### Overview

The use of search engines and websites to seek medical information is continually growing. As the human life span increases due to novel medical interventions and technologies, more and more individuals are developing chronic illnesses. In this study, we distinguish between general forums where one can ask questions about health conditions, general social networks where the user is identified and “converses” with friends (eg, Facebook), and specific forums related to a unique interest group such as a social health network. The essential difference is that a social network includes many different features (such as blogs and live chats), whereas a forum uses only one of the tools found in an interactive health site. Although information about the usefulness of medical social networks for certain conditions exists, very little attention has been paid to how patient involvement in an online health-related social network is related to how they manage their health-related conditions. Due to the increase in social health networks, this study evaluated patient activation in this context.

Patient activation describes how much a patient is involved in his or her health care. Online health-related social networks provide patients with information about their disease and the ability to learn more about it by interacting with others with a similar problem. We looked at the concepts of patient activation and online health site use in an effort to determine what factors influence how useful patients perceive the site to be. This study aimed to explore the combined effects of patient activation and involvement in an online health-related social network on the perceived usefulness of such websites. By increasing our knowledge regarding the factors that underlie how patients perceive and gain information from the Internet, we can continue to reshape and develop online medical and health information in ways that are compatible with the needs of the target users.

### Background

The use of online support groups has increased dramatically in recent years. In 1998, over 50 million American adults turned to the Internet to obtain health information. By 2005, that number had increased to 117 million and in the course of a single year—from 2009 to 2010—this number rose from 154 million to 175 million [1]. Statistics from 2009 indicate that an estimated 58% of all Internet users consulted the Web for health purposes, 61% of American adults looked online for health information, and roughly one-third accessed social media related to health [2].

Individuals draw on many different sources of information for health-related decisions. The Internet adds to available resources by offering specialized blogs and by allowing individuals to seek advice from question-and-answer type health forums hosted by experts and from interactive social health networks. Participation in a social health network can help people feel connected to others by giving them the opportunity to offer and obtain information about their diseases, although individuals are more likely to consume information than they are to contribute to the dialogue [3].

Medical online support groups are designed to improve individuals’ understanding of their health conditions, change their health behaviors, and enhance their ability to manage a chronic health condition. They can also enhance self-confidence by providing increased emotional support and by enabling members to better manage their diseases [4-10]. Online health-related social networks have changed the patient-physician relationship and have benefited both parties: patients receive external support and information, whereas clinicians gain increased accessibility to new ideas and alternative therapies and approaches from their patients [11-14].

Patients who are more likely to search online for health care information include women [15,16], those with a higher education, a chronic health condition, more years of Internet experience, and those with broadband access [17,18]. Associations between factors such as income and age with online health-related information seeking are less consistent [19-21].

### Patient Activation

The underlying theory behind the Patient Activation Measure is that “*Activation* refers to having the capability and the willingness to take on the role of managing one’s own health and health care” [22]. “Patient activation” describes the extent to which individuals are able to manage their own health care. Hibbard and her colleagues [23,24] conceptualized patient activation as encompassing a range of elements important to self-management that extend beyond any single health behavior. As a measure of this concept, they developed the Patient Activation Measure (PAM), a broad construct that encompasses self-efficacy, behavior, and knowledge. It can predict a variety of behaviors including healthy behavior, preventive care measures, disease-specific self-care behavior, and information seeking [22]. The PAM is a 13-item psychometric tool with a 4-level model of health-related behaviors that measures the latent construct of patient activation. It captures the degree to which patients have the beliefs, knowledge, and skills to “manage their condition(s), collaborate with their providers, maintain their health, and access appropriate and high-quality care” [24,25].

Studies have found that supportive social environments lead to increased activation levels [26]. Higher activation scores have also been linked with sociodemographic factors of female gender, younger age, and higher education or income levels.

Several studies have explored the relationship between patient activation and individuals with illnesses such as HIV [27], asthma [28], diabetes [29], inflammatory bowel disease [30], spine surgery [31], multiple sclerosis [32], and mental illnesses [33]. Research has demonstrated that increasing patients’ self-management with regard to their illness increased their PAM scores, which in turn was connected with more favorable health outcomes. Additionally, higher PAM scores were associated with greater degrees of optimism, hope, and control [31].

Activated patients strive to understand their health conditions, viewing problems as challenges, and displaying confidence with regard to their positive resolution [25,34]. Acquiring the attributes of self-management is a gradual process that involves the attainment of knowledge and problem-solving skills that enable individuals to confidently engage in decision-making and actions aimed at managing their chronic health condition more effectively [35-38]. Activated patients are more likely to adhere to behaviors that help control their symptoms and the progression of their disease [32]. Higher activation has been associated with less use of health care services [39].

Only two previous studies examined the effect of a Web-based intervention, similar to our site, on patient activation among individuals with a chronic health condition. One, which included patients with diabetes, asthma, or hypertension, found that the Web-based intervention had a positive effect on the patient activation levels of participants in the intervention group [40]. Likewise, a study of patients with type 2 diabetes found that patient activation improved after participation in the online diabetes self-management program [41]. However, we were unable to find a previous study that specifically examined patient activation measures in reference to use of an online health-related social network. Most existing information pertains to simple online medical question-and-answer sites, which provide a more limited scope than interactive websites that offer a range of services including blogs, patient information exchanges, and contact with physicians.

Thus, the purpose of this study was to further evaluate the effects and benefits of participating in an online health-related social network on patient activation and to determine which variables predict perceived usefulness of the site. We hypothesized that patients with higher activation scores would tend to follow the information provided by an interactive website and consequently perceive it as more useful. As patients who use an interactive website frequently become adept at obtaining relevant information quickly and efficiently, we predicted that high levels of website involvement would also predict perceived website usefulness.

## Methods

### The Platform

**Figure 1:**
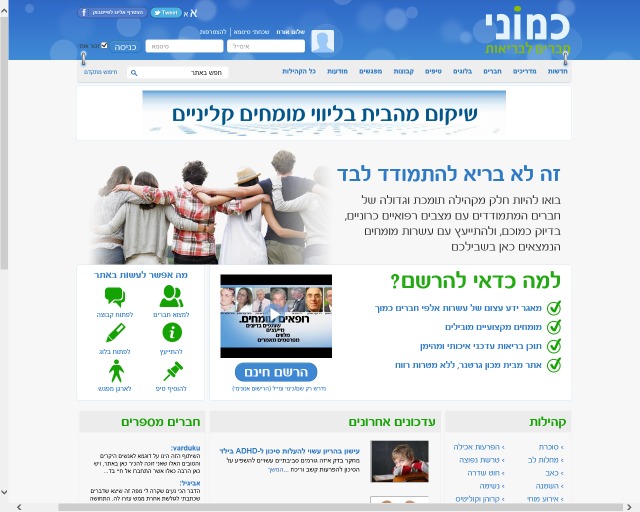
Screenshot of the Camoni website.

Camoni is the first Hebrew-language, nonprofit, online, social network that is targeted to individuals with chronic conditions and assists them in finding others facing similar health issues [42]. The Hebrew word “camoni” means “like me”. The Camoni site is comprised of 16 communities, defined according to the health conditions of diabetes, chronic pain, heart disease, hypertension, obesity, eating disorders, multiple sclerosis, spinal injury, lung disease, kidney disease, stroke, osteoporosis, Crohn’s disease, cancer, obesity, and depression. Figure 1 shows a page from the Camoni website. Each community is headed by a medical expert. Camoni offers advice, the opportunity to consult with experts, and the chance to converse with other patients who face the same health condition. The site includes blogs, forums, support groups, internal mail, and chats. It also explains each health condition, its diagnosis, and offers practical advice on how to maintain one’s health and cope with the disease. Registration is required for active participation on the site, which is open to all. Camoni enables people to share, to learn and gain encouragement from each other, and to provide advice based on their own experiences. Since its launch in August 2009, Camoni has grown exponentially and today has thousands of registered users. This study focused on the five largest communities: diabetes, heart disease, kidney disease, spinal injury, and depression/anxiety.

### Recruitment

This cross-sectional study is based on a single sampling time. Recruitment was conducted during a 4-month period that began in February 2012 and continued through May 2012. The questionnaire was accessible through Google Docs (see Multimedia Appendix 1 for an English translation). An invitation to participate and a link were available on the Camoni home page of each of the chosen disease communities (diabetes, heart disease, spinal injury, kidney disease, and depression/anxiety). During the three-month recruitment period, reminders were placed in the monthly newsletter sent to all Camoni participants who had not declined the option. An individual could only answer once.

### Instruments

#### Demographic and Health Characteristics

Respondents (n=296) were asked to provide information about basic demographic variables such as age group, gender, income, and diagnosis, as well as information regarding their use of the website. Descriptive information regarding the cohort is shown in Table 1.

**Table 1:** Demographics and health statistics of the cohort^a^ (n=296).

Variable		n	%
**Gender**			
	Male	135	45.6
	Female	161	54.4
**Age range, years**			
	20-29	23	7.8
	30-39	20	6.8
	40-49	46	15.5
	50-64	140	47.3
	65+	64	21.6
**Income**			
	Below average	143	48.3
	Average	59	19.9
	Above average	64	21.6
**Illness**			
	Diabetes	115	38.9
	Heart	31	10.5
	Kidney	25	8.4
	Spine	59	19.9
	Depression/Anxiety	53	17.9
**Number of illnesses**			
	1	95	32.1
	2	143	48.3
	3+	58	19.6
**Duration of illness**			
	Less than 6 months	8	2.7
	6-12 months	13	4.4
	1-2 years	24	8.1
	2-4 years	50	16.9
	Over 4 years	193	65.2
**Duration of activity on website**			
	Less than 6 months	104	35.1
	6-12 months	73	24.7
	1-2 years	85	28.7
	2-3 years	32	10.8
**Frequency of activity on website**			
	Every day	30	10.1
	1-2 times a week	89	30.1
	1-2 times every 2 weeks	55	18.6
	1-2 times a month	41	13.9
	Once a month	66	22.3

^a^Does not include missing values.

#### Perceived Usefulness of Online Groups

Perceived usefulness of the website was measured by 13 items on a scale ranging from 1 (“very little”) to 5 (“very much”). Possible scores ranged from 0 to 65. It is a measure of the extent to which participants found the website helpful and informative (eg, “Conversations/chats with other surfers on Camoni helped me”; “Because of Camoni, I changed the type of treatment”; “On Camoni, I found new information about a treatment type”; “I use the information I obtained when I visit my doctor”). Cronbach alpha was high (.93), and a principal component factor analysis with varimax rotation yielded one main factor (Eigenvalue =7.02), which accounted for 54% of the variance. Accordingly, a mean perceived usefulness score was calculated for each respondent.

#### Active Involvement in Online Groups

Active involvement was measured by a 9-item scale ranging from 1 (“very little”) to 5 (“very much”), including items measuring the extent to which respondents participated in the website components (eg, reading/writing articles, reading/writing blogs, chats). Possible scores ranged from 0 to 45. Here too, Cronbach alpha was high (.84) and a principal component factor analysis with varimax rotation yielded one main factor (Eigenvalue =4.15), which accounted for 52% of the variance. Accordingly, a mean score of active involvement was calculated for each participant.

#### Patient Activation

We used the 13-item patient activation measure (PAM-13) to quantify patient activation. The PAM-13 is designed to elicit responses about an individual’s attitudes toward knowledge, skills, and confidence in self-managing health [23,25]. The scale is based on the Guttman technique with items ordered according to level of difficulty (eg, “When all is said and done, I am the person who is responsible for taking care of my health”). Patients are required to indicate their agreement with each item on a scale ranging from 1 (“Disagree strongly”) to 4 (“Agree strongly”), or to indicate that the item is not applicable to them. Possible scores ranged from 0 to 100. Cronbach alpha for this scale was .86. Accordingly, a mean patient activation score was calculated for each participant.

#### Data Analysis

Data were analyzed with the statistical software package SPSS 20. Age and gender differences were examined by analyses of variance (ANOVA), in which both were independent variables and active involvement, perceived usefulness, and patient activation were the dependent variables. The contribution of independent variables to perceived website usefulness was examined by a hierarchical regression. The first step included demographic variables (gender, age, income, duration and frequency of activity in the website, duration of illness) and the second step included the number and type of illnesses an individual has (diabetes, heart disease, kidney disease, spinal injury, and anxiety/depression). The third step included active involvement in the website and the patient activation measure.

## Results

### Active Involvement

Main effects for age (*F*_9,260_=2.16, *P*=.07, *η^2^*=.03) and gender (*F*_9,260_=1.51, *P*=.22, *η^2^*=.01) were not significant. However, a significant interaction of age × gender was found, (*F*_9,260_=3.36, *P*=.01, *η^2^*=.05). In order to discover the root of the interaction, we conducted two separate ANOVA for males and females, in which the dependent variable was active involvement and the independent variable was age. The ANOVA revealed a significant effect of age for male participants (*F*_4,120_=4.02, *P*=.01, *η^2^*=.12), but not for females (*F*_4,140_=0.64, *P*=.637, *η^2^*=.01). Scheffe’s post hoc tests demonstrated that men 30-39 years of age scored significantly higher in active involvement (mean 4.16, SD 0.99) than those 40-49 years (mean 2.62, SD 1.15; *P*=.03), 50-64 years (mean 2.46, SD 0.99; *P*=.004), and 65+ years (mean 2.58, SD 0.90; *P*=.01).

### Perceived Usefulness

A significant main effect of age was found, (*F*_9,261_=3.22, *P*=.01, *η^2^*=.05). Pairwise comparisons demonstrated that individuals 20-29 years of age scored significantly higher in perceived usefulness (mean 2.26, SD 1.24) than those aged 50-64 years (mean 1.43, SD 1.18; *P*=.04) and 65+ years (mean 1.38, SD 1.00; *P*=.049). Gender was not significant (*F*_9,261_=2.19, *P*=.13, *η* ^2^=.01).

### Patient Activation Measure

A significant main effect of age was found (*F*_9,279_= 4.41, *P*=.003, *η^2^*=.06). Pairwise comparisons demonstrated that individuals aged 20-29 years scored significantly lower on the PAM-13 scale (mean 48.44, SD 21.25) than those aged 30-39 years (mean 62.28, SD 19.78; *P*=.01) and 50-64 years (mean 57.50, SD 17.66; *P*=.049). Gender was not significant (*F*_9,283_=0.17, *P*=.76, *η^2^*=.01).

### Factors Contributing to Perceived Usefulness

Several variables were significant predictors for perceived usefulness (see Table 2). Age was found to be a negative predictor: younger age was indicative of higher perceived usefulness. Active involvement was a positive predictor, thereby demonstrating that such website involvement promotes perceived usefulness. More interesting, however, was the negative connection between PAM scores and perceived usefulness, as individuals who take a less active role in their care found that the website was more useful and provided them with information regarding their ailment. Additionally, a near-significant association (*P*=.06) between higher frequency of activity and an increase in perceived usefulness was found.

**Table 2:** Hierarchal multiple regression analysis predicting perceived website usefulness (n=296).

							Step 1			Step 2		Step 3	
Predictor					*ΔR^2^*		*B*		*P*	*B*	*P*	*B*	*P*
**Step 1**					.121^b^								
		Gender					−.33		.04^a^	−.29	.08	−.26	.08
		Age					−.23		.001^b^	−.26	.001^b^	−.26	.001^b^
		Income					−.04		.53	−.05	.44	−.01	.78
		Duration of activity in website					.08		.27	.10	.18	.09	.17
		Frequency of activity in website					−.21		.001^b^	−.21	.001^b^	.10	.06
		Duration of illness					.05		.54	.02	.81	−.05	.48
**Step 2**					.036								
		Diabetes								−.20	.48	−.12	.65
		Heart								.61	.08	.61	.07
		Kidney								−.05	.85	−.07	.72
		Spine								−.13	.64	−.13	.61
		Depression/anxiety								−.31	.32	−.40	.17
		Number of illnesses								.02	.93	.02	.96
**Step 3**					.142^b^								
		Active involvement in site										.45	.001^b^
		Patient Activation Measure										−.01	.04^a^
Total *R* ^*2*^					.299								

^a^ *P*<.05

^b^ *P*<.001

## Discussion

### Principal Findings

To the best of our knowledge, this is the first study to examine how a patient’s degree of active involvement in managing their health and their participation in an online social health forum affected the perceived usefulness of such a website.

Online social groups have the broadest reach and impact when the target population is younger [43,44]. Although chronic diseases are more prevalent among older individuals, fewer use the Internet as a source of information. However, chronic illness is a growing problem among younger individuals as well [45,46]. The increase in the number of younger, computer literate individuals with chronic illness is contributing to the growth of websites like Camoni.

We found that individuals 20 to 29 years of age reported higher perceived usefulness of the website and its contents compared to those over the age of 50. The relatively low perceived website usefulness and lesser involvement among older adults may be a consequence of difficulty in accessing relevant information online. We suggest that younger people experience greater perceived usefulness because they use the Internet more [16] and enjoy sharing personal aspects of their life on the Internet (for example, Facebook). They also belong to a generation that is used to immediate results (instant messaging, instant meals, instant gratification, etc.) and therefore prefer to obtain health information online rather than wait for a physician consultation. In the past, authoritative health information originated within the physician’s office [47,48]. This is still true for many older individuals. We see a need for a longitudinal study to determine the effects of information gathering and social support from online communities and from health forums on patient-physician relationships.

Additionally, individuals 20 to 29 years of age scored significantly lower on the PAM-13 scale than those 30 to 39 and 50 to 64 years of age. We believe that these two age groups are more likely to seek health information. Individuals 30 to 39 years of age are often parents of young children and therefore seek health information to preserve their health and well-being as parents and as care providers. They also might seek health information regarding their children. The 50 to 64 age group is likely to demonstrate greater activation because they are beginning to encounter more chronic illness and are interested in preserving their health.

Unlike other studies [3], we found more men using the website than women. It is possible, since we found that men were more interested in using the site to obtain information and improve their health, they use Camoni to learn more about their condition rather than for peer support, as they tend to be less inclined to discuss personal issues and ask for advice than women are.

In this study, both younger age and active involvement in the site were indicative of higher perceived website value. Of interest was the negative relationship between PAM scores and perceived usefulness, as individuals who were less active in their care found that Camoni was more useful compared to those with higher PAM scores. We believe that this finding might have important ramifications regarding patients’ ability to actively participate in their own health care management. Perhaps those with lower PAM scores have more to gain from the website, both in terms of medical information and the experiences of others. Therefore, we are planning a study that will measure changes in PAM among new participants in Camoni. Several authors also found that Web-based interventions benefited participants more at the earliest stages of patient activation. They also suggested that these patients have the most to gain from these websites [40,49].

We had anticipated that higher PAM levels would predict greater perceived usefulness, because, by definition, a high PAM level means that the individual is involved in his or her care and will therefore seek a variety of resources, one of which is a health-related social network. Unexpectedly, we found that low PAM scores predicted greater perceived website usefulness. We suspect that those who newly enter the site are at an early stage in their illness and are at a low PAM level. They are more open and willing to participate and receive information regarding their health status through the site. However, at this stage, they still are not ready to actively participate in their self-care and comply with medical advice. Their activation is only at the level of interacting with a peer group with similar problems, who “know what they are going through”. We suggest that a future study that follows these individuals would find that PAM scores increase from the time of their initial participation.

### Limitations of the Present Study

This study sampled Israelis and was conducted in the Hebrew language. Therefore, it should be expanded to include other nationalities. Moreover, as with many studies that concern Web activity, the sample used was self-selected; consequently, it might be difficult to infer causation from the results. Additionally, this study included five disease groups, but necessarily excluded others. We did not have information about other sources of knowledge or support that the participants might have accessed. For example, we do not know about additional medical consultations, family members who provided support, other forums or Internet sites where they received information, or avenues such as medical books or pamphlets.

### Conclusions

The study results suggest that participation in an online health-related social network can be particularly helpful to individuals who have lower levels of patient activation. As these individuals might have less exposure to other sources of online medical information, they find specialized social health networks such as Camoni more useful. We can assume that such individuals are able to benefit from others seeking similar information. It may well be that once they observe how others weigh information from different sources and give support to each other, they are encouraged to do the same and subsequently take a more active role in managing their own health. Thus, we anticipate that this modeling behavior might lead to an increase in PAM measures, which in turn, will be related to better compliance with medical regimens. We are implementing a prospective study to test this theory.

## Multimedia Appendix 1

English translation of the study questionnaire.

## Abbreviations

PAM: patient activation measure
