# Supplementary material for: Online Activity and Participation in Treatment Affects the Perceived Efficacy of Social Health Networks Among Patients With Chronic Illness
Source: J Med Internet Res. 2014 Jan 10;16(1):e12. doi: 10.2196/jmir.2630 (PMC3906665; doi:10.2196/jmir.2630)
Supplement: Supplementary file 1 [file jmir_v16i1e12_app1.pdf]

Dear Participant,

Thank you for agreeing to participate in this research study, conducted by the Department of Management at Bar Ilan University, Israel. The study examines various aspects and characteristics of patients with a chronic illness who use the online health-related social network "Camoni". The information you provide will be used for research purposes only, and you will not be identified in any way. Please answer all questions to the best of your ability, feelings, and knowledge.

You will first be asked to provide general information about yourself and then you will be asked about your experiences with your illness relating to Camoni.

<u>Part I: Demographic information</u>

In the following questions, please indicate the correct information:

1. Gender:

    a. Male

    b. Female

2. Age-Group:

    a. 20-29

    b. 30-39

    c. 40-49

    d. 50-64

    e. 65+

3. The average monthly income in Israel is 8,190 NIS. How does your income compare to this figure?

    a. Below average

b. Average

c. Above average

4. Of the following medical conditions, which is/are the one/s you are currently dealing with? (***Please note***: you may mark more than one medical condition)

   a. Diabetes

   b. Heart condition

   c. Kidney disease

   d. Spine injury

   e. Depression/anxiety

5. How long have you been afflicted with the condition (if you marked several conditions, please refer to the one with the longest duration)

   a. Less than 6 months

   b. 6-12 months

   c. 1-2 years

   d. 2-4 years

   e. Over 4 years

6. How long have you been a member of Camoni?

   a. Less than 6 months

   b. 6-12 months

   c. 1-2 years

   d. 2-3 years

7. How often do you enter the Camoni website?

   a. Every day

   b. 1-2 times a week

   c. 1-2 times every two weeks

   d. 1-2 times a month

   e. Once a month

Part II

Please rate the following statements and indicate your agreement with each statement according to the given scale:

	Question	Not at all	Very little	A little	Neutral	A lot	Very much
1	I tend to discuss my medical condition with members of Camoni	0	1	2	3	4	5
2	I find it useful to talk to members of Camoni	0	1	2	3	4	5
3	I tend to consult with the medical experts of Camoni	0	1	2	3	4	5
4	Talking to the medical experts of Camoni is beneficial to me	0	1	2	3	4	5
5	The information I find in Camoni helps me to improve my health habits	0	1	2	3	4	5
6	After Camoni, I changed my choice of treatment	0	1	2	3	4	5
7	In Camoni, I learned of a medicine I was not aware of	0	1	2	3	4	5
8	In Camoni, I received new information about treatment methods	0	1	2	3	4	5
9	Camoni is a venue for health issues	0	1	2	3	4	5
10	I use the information I receive in Camoni when I visit my physician	0	1	2	3	4	5
11	Since joining Camoni, I feel more involved in making medical decisions relating to my condition	0	1	2	3	4	5
12	In Camoni, I received support which helped me to deal with my condition	0	1	2	3	4	5
13	Camoni helped me alleviate feelings of loneliness	0	1	2	3	4	5

Now, please read the following list of various possible activities on Camoni and rate the extent to which you engage in these online activities according to the scale given

	Factor	N/A	Very little	A little	Mediocre	Medium	A lot	Very much
1	Receiving information from health forums	0	1	2	3		4	5
2	Reading articles	0	1	2	3		4	5
3	Writing articles	0	1	2	3		4	5
4	Opening a blog	0	1	2	3		4	5
5	Reading blogs	0	1	2	3		4	5
6	Chats	0	1	2	3		4	5
7	Personal messages	0	1	2	3		4	5
8	Joining a group	0	1	2	3		4	5
9	Asking questions in health forums	0	1	2	3		4	5

Below are some statements that people sometimes make when they talk about their health. Please indicate how much you agree or disagree with each statement as it applies to you personally by circling your answer. Your answers should be what is true for you and not just what you think the doctor wants you to say. If the statement does not apply to you, circle N/A.

	Statement	N/A	Disagree Strongly	Disagree	Agree	Agree Strongly
1	When all is said and done, I am the person who is responsible for taking care of my health	0	1	2	3	4
2	Taking an active role in my own healthcare is the most important thing that affects my health	0	1	2	3	4
3	I am confident I can help prevent or reduce problems associated with my health	0	1	2	3	4
4	I know what each of my prescribed medications do	0	1	2	3	4
5	I am confident that I can tell whether I need to go to the doctor or whether I can take care of a health problem myself	0	1	2	3	4
6	I am confident that I can tell a doctor concerns I have, even when he or she does not ask	0	1	2	3	4
7	I am confident that I can follow through on medical treatments I may need to do at home	0	1	2	3	4
8	I understand my health problems and what causes them	0	1	2	3	4
9	I know what treatments are available for my health problems	0	1	2	3	4
10	I have been able to maintain lifestyle changes, like eating right or exercising	0	1	2	3	4
11	I know how to prevent problems with my health	0	1	2	3	4
12	I am confident I can figure out solutions when new problems arise with my health	0	1	2	3	4

13	I am confident that I can maintain lifestyle changes, like eating right and exercising, even during times of stress	0	1	2	3	4
